# Detailing Protein-Bound Uremic Toxin Interaction Mechanisms with Human Serum Albumin in the Pursuit of Designing Competitive Binders

**DOI:** 10.3390/ijms24087452

**Published:** 2023-04-18

**Authors:** Vida Dehghan Niestanak, Larry D. Unsworth

**Affiliations:** 1 Department of Biomedical Engineering, University of Alberta, Edmonton, AB T6G 2G4, Canada; vida1@ualberta.ca; 2 Department of Chemical and Materials Engineering, University of Alberta, Edmonton, AB T6G 1H9, Canada

**Keywords:** chronic kidney disease, protein-bound uremic toxins, human serum albumin, binding affinity

## Abstract

Chronic kidney disease is the gradual progression of kidney dysfunction and involves numerous co-morbidities, one of the leading causes of mortality. One of the primary complications of kidney dysfunction is the accumulation of toxins in the bloodstream, particularly protein-bound uremic toxins (PBUTs), which have a high affinity for plasma proteins. The buildup of PBUTs in the blood reduces the effectiveness of conventional treatments, such as hemodialysis. Moreover, PBUTs can bind to blood plasma proteins, such as human serum albumin, alter their conformational structure, block binding sites for other valuable endogenous or exogenous substances, and exacerbate the co-existing medical conditions associated with kidney disease. The inadequacy of hemodialysis in clearing PBUTs underscores the significance of researching the binding mechanisms of these toxins with blood proteins, with a critical analysis of the methods used to obtain this information. Here, we gathered the available data on the binding of indoxyl sulfate, p-cresyl sulfate, indole 3-acetic acid, hippuric acid, 3-carboxyl-4-methyl-5-propyl-2-furan propanoic acid, and phenylacetic acid to human serum albumin and reviewed the common techniques used to investigate the thermodynamics and structure of the PBUT–albumin interaction. These findings can be critical in investigating molecules that can displace toxins on HSA and improve their clearance by standard dialysis or designing adsorbents with greater affinity for PBUTs than HSA.

## 1. Introduction

Kidney dysfunction ultimately leads to the retention of otherwise cleared metabolites (i.e., uremic toxins [1]) in the blood compartment. The increased retention of these uremic toxins further exacerbates kidney health and contributes to the development of chronic kidney disease (CKD), as well as being correlated with other major co-morbidities [2]. CKD is among the top 20 fatal diseases recognized globally, and by 2040 it is estimated to be the fifth largest health issue faced worldwide [3,4]. According to the Canadian Institute for Health Information [5], the number of Canadians who start renal replacement therapy increases by 1.1% annually. Within two decades, twice as many people will be prescribed dialysis due to renal dysfunction in Canada. Worldwide, more than 10% of the population suffers from CKD, accounting for 1.2 million deaths and 28 million years of life lost each year [3].

**Figure 1:**
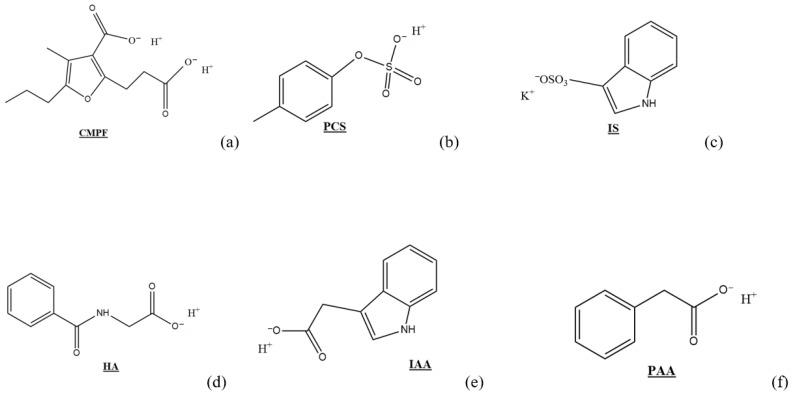
Chemical structure and charge location for (**a**) 3−carboxyl−4−methyl−5−propyl−2−furan propanoic acid (CMPF), (**b**) p−cresyl sulfate (PCS), (**c**) indoxyl sulfate (IS), (**d**) hippuric acid (HA), (**e**) indole−3−acetic acid (IAA), and (**f**) phenylacetic acid (PAA).

Hemodialysis is the conventional method for clearing uremic toxins from the blood [1]. However, despite decades of research, the average life expectancy of CKD patients receiving hemodialysis has neither increased beyond three years nor has the incidence or severity of CKD co-morbidities been significantly reduced [6,7]. Protein-bound uremic toxins (PBUTs) are thought to play a crucial role in the health of patients with kidney dysfunction. These small molecular weight toxins (Mw < 500 Da) become bound to human blood proteins (i.e., albumin) and, consequently, cannot be cleared from the blood compartment using membrane-based hemodialysis (see Table 1, Figure 1) [1,8]. Co-morbidities associated with the accumulation of known PBUTs, namely indoxyl sulfate (IS), p-cresyl sulfate (PCS), indole 3-acetic acid (IAA), hippuric acid (HA), 3-carboxyl-4-methyl-5-propyl-2-furan propanoic acid (CMPF), and phenylacetic acid (PAA), are summarized in Table 1. The difference between the blood concentration of these molecules in normal kidneys or those with failing kidneys can be as high as 400 times. That said, the interaction mechanisms between well-known uremic toxins and blood proteins remain largely ill-defined. Elucidating these mechanisms will allow innovation in developing means to remove these toxins, which may include competitive inhibitors and adsorption-based techniques: the former leading to increased clearance efficiency using membrane dialysis, the latter focusing on directly capturing the toxins [1,4,9].

Human serum albumin (HSA) is the most abundant blood protein (~60% of total protein, at 0.6 mM) with a net negative charge of −19, at a pH of 7.4, an isoelectric point of 5.4, 585 amino acids, and three structurally similar, homologous α-helical domains (I, II, and III), each with ten helices divided into 6-helix and 4-helix subdomains (A and B) [10,11,12]. HSA is crucial in binding organic and inorganic molecules, including hydrophobic and anionic substances, either covalently or non-covalently. In patients with CKD, HSA binding capacity is significantly lowered [13,14]. This is thought to occur due to the saturation of HSA binding sites by uremic toxins and HSA conformational changes during post-translational modification such as oxidation, glycosylation, and carbamylation [14,15]. Although ligand–HSA interactions are complicated by both specific and non-specific binding sites that may be involved, depending on the uremic toxin and concentration, it has been determined that PBUT bindings essentially occur at Sudlow’s site I (subdomain IIA) and site II (subdomain IIIA), a common location where aromatics and heterocyclic ligands are known to bind [10,11]. Bulky heterocyclic anions charged in the middle generally bond to site I, while aromatic carboxylic acids with charges at one end are more likely to bind to site II [16]. Furthermore, ligand binding with subdomains IB, IIA, and IIIB and these subdomains’ interface is vital as these regions hold seven binding sites for fatty acids [11]. Davilas A. et al. [13] highlighted that since there is a common interface between site I and site II, the binding of uremic toxins to each subdomain likely results in conformational and binding behavior changes, and HSA structure changes become more significant while studying several PBUT competitions for the same binding site on HSA.

**Table 1:** Physical properties of protein-bound uremic toxins, significance in CKD patients, and major co-morbidities caused by the accumulation in the blood compartment.

PBUT	Chemical Group	Molecular Weight (Da)	Average Normal Concentration (mg/L)	Average Uremic Concentration (mg/L)	Average Hemodialysis Reduction (%)	Major Co-Morbidities	Refs.
3-Carboxyl-4-Methyl-5-Propyl-2-Furan Propanoic Acid (CMPF)	Furan Dicarboxylic Acids	240	4.6 (±1.80)	25.95 (±10.23)	20	Altered drug pharmacokinetics, increased intestinal permeability, neurological abnormalities, renal tubule damage, anemia	[4,17,18,19,20]
Indoxyl Sulfate (IS)	Indoles	212	0.53 (±0.29)	3.83 (±2.46)	36	Altered drug pharmacokinetics, bone and cardiovascular diseases, oxidative stress, muscle weakness, endothelial cell, wound repair inhibition	[15,17,18,21,22]
p-Cresyl sulfate (PCS)	Phenols	187	0.08 (±0.09)	2.60 (±5.10)	31	All-cause mortality, oxidative stress, inflammation, cardiovascular disease	[17,18,22,23]
Hippuric Acid (HA)	Hippurates	179	3.00 (±2.00)	109.43 (±64.66)	67	Endothelial dysfunction, renal tubule damage	[17,18,24,25]
Phenylacetic Acid (PAA)	Others	136	1.4	474.64 (±44.88)	35	All-cause mortality, oxidative stress, inflammation, atherosclerosis, cardiovascular disease, increased immunodeficiency	[17,18,26,27,28]
Indole-3-acetic acid (IAA)	Indoles	174	0.50 (±0.30)	1.26 (±0.83)	45	Altered drug pharmacokinetics, bone and cardiovascular diseases, oxidative stress, muscle weakness, neuropathy, cytotoxicity, cognitive impairment	[4,17,18,29,30]

Herein, we specifically review the experimental techniques used in the context of understanding the binding of PBUTs with HSA. The accuracy of the standard methods applied to obtain thermodynamic data and investigate protein structure changes during the binding event will be discussed. Additionally, we review the literature regarding PBUT binding to HSA, highlighting relevant physicochemical properties, binding affinities, and binding mechanisms. This information is critical for developing novel pathways to remove these uremic toxins from the blood compartment.

## 2. Experimental Techniques Commonly Employed

Isothermal Titration Calorimetry (ITC): An ITC instrument consists of a thermally equilibrated syringe, sample, and reference cells [31,32]. The interaction between the sample and sequentially injected titrant causes temperature changes between the reference and experimental cell, where the energy required to balance these changes is integrated to obtain changes in heat as a function of titrant concentration. This information can be used to describe the complete thermodynamic profile of molecular interaction, namely the association binding constant (*K_a_*), enthalpy change (ΔH), stoichiometry (*n*), Gibb’s free energy (ΔG), and entropy change (ΔS) [31,33]. Moreover, the heat of capacity (ΔC_p_) can be obtained by changing the temperature to study the binding event [32,34,35].

In order to collect accurate isotherms, protein and ligand concentrations need to be studied within *c*-values between 1 and 1000 [36], as determined using the expected *K_a_* and the number of ligands (*n*) in the chamber:

(1) $$c=nK_{a}$$

Analysis of collected isotherms for determining thermodynamic parameters relies on several underlying assumptions. Firstly, the binding reaction is assumed to be reversible and in equilibrium. Although this assumption is acceptable for most binding reactions, one should note that allosteric changes caused by binding may directly affect the reversibility of the binding event itself [31,32,35,36,37]. For example, PBUT buildup in the blood has been shown to affect plasma protein conformation, where HSA α-helicity drops from its native 54–56% to 8% when PCS is present at physiological conditions for CKD patients, with the subsequent strongest response by IS, followed by HA, IAA, and CMPF [32,38]. IS and PCS presence yielded an increase in the β-sheet content of HSA by 15.6% and 23%, respectively. This is a clear case where the protein’s conformation changes may affect the PBUT binding characteristics. Secondly, all molecules remain soluble, and the attributed ΔH of dissolution is detected and subtracted from the total ΔH measured [36]. Finally, the macromolecule solution is assumed to be ideal, where no macromolecule–macromolecule/co-solute interactions occur. However, this is practically impossible as macromolecules interact with co-solutes and each other by direct binding and indirect modification of vicinal hydration layers [36]. Hence, an additional blank experiment, which can be either a buffer-to-protein solution or toxin-to-buffer titration, is set. This isotherm is subtracted from the ligand–protein data to remove any heat generated/absorbed in secondary interactions, hydration layer modifications, and mixing into the solution [31,32]. With this in mind, it is possible to model the isotherms collected for these molecular interactions as outlined below.

*ITC Model 1: The Single Set of Independent Binding Sites Model:* This model is used when only one binding site is assumed to be accessible to the PBUT for HSA. The model is derived from the Langmuir adsorption theorem, which is based on the equilibrium between the number of available and occupied binding sites on protein in a solution and can also be correlated to the concentration of free titrant molecules (i.e., uremic toxins) in the media [31,37,39]. As there is often more than one binding region, the fittings provide thermodynamic insights into the overall interaction of the protein with the toxin [37]. That said, even if the model used has a good fit on ITC isotherms, it does not necessarily mean that it represents the actual situation; care must be taken to understand the effect the binding has on the protein as a function of concentration. Moreover, the returned ΔH and further energy calculations are usually considered much more accurate than the returned number of binding sites (*n*) [31]. Nevertheless, the independent model was found to be most appropriate for IS and PCS since these uremic toxins predominantly interact with a single binding site on HSA (see below) [13,32,40].

*ITC Model 2: The Sequential Binding Sites Model:* This model is used for more complicated interaction events where more than one site on HSA accepts the toxin. Each binding site’s thermodynamic properties are obtained based on the equilibrium stage sequences. It should be noted that this sequence is not necessarily linked with the binding affinity of the toxin to the region on HSA [37]. Zaidi et al. found the sequential binding model to be the best fit for the ITC results of the HA and HSA interaction. However, they observed deviation in ΔC from the literature, which may result from changes in protein structure as a function of solution temperature. They pointed out that the ITC result is limited to whether the interaction or temperature change is causing protein conformational alterations. Hence, it is necessary to combine ITC results with an analysis of HSA conformation under similar solution conditions [35].

In most cases, there is more than one binding site available on HSA for uremic toxins, and in sequential binding, it is assumed that previous binding events affect subsequent interactions, which seems more likely because toxin binding may influence HSA structure [41]. If the binding sites are identical (i.e., similar values for K and ΔH), the isotherm is commonly described using the single independent site model. Indeed, prior investigation on the structure and chemistry of the toxins will be beneficial in preventing misinterpretation of the data [31]. For instance, at low PAA molar ratios, independent and sequential binding site models were fitted ideally on PAA-HSA ITC results. Yu et al. showed that for molar ratios higher than 15 n(PAA)/n(HSA), these two models deviate, and the sequential model seems to be a better candidate, indicating that at a threshold concentration, two binding sites on HSA arise for PAA [37].

*ITC Model 3: The Competitive Binding Model:* In addition to understanding how toxins interact with HSA, competitive binding mechanisms can be elucidated using ITC isotherms by fitting competitive binding models. An example is when Li et al. investigated the displacement of IS and PCS from HSA by either warfarin or ibuprofen. These drugs were considered ligands for the toxin–HSA complex. The blank isotherms were subtracted from the displacement results for the toxin–HSA complex and buffer, in this case. In the final isotherm profile, both endothermic and exothermic injections are usually seen, which are claimed to result from displacing the toxin by the binding competitor. The prior ligand–protein ITC isotherms and thermodynamic profile provide insights into further interpreting the competitive binding results [32]. Multiple independent binding site models can also be used in similar cases where more than one ligand interacts with the protein. The binding constants and energy involved are independently obtained for each toxin regarding their high-affinity site on HSA [37].

The advantage of ITC over other thermodynamic techniques is the ease of collecting data in the least number of experiments and that no probe or ligand immobilization at a surface is required [31,32]. Due to its extensive measurement range, *K_a_* from 10^3^ to 10^9^ M^−1^ [31], ITC is utilized in various applications for different molecule sizes down to nanoparticles and small solutes and solvents [33]. While other techniques such as UV spectroscopy, surface plasmon resonance (SPR), or quartz crystal microbalance (QCM) may be more affordable, they do not possess high sensitivity or resolution. Moreover, using these techniques, the samples must undergo adaptations, such as immobilization [31,42].

ITC data can suffer due to its sensitivity to temperature, pH, and other environmental factors [36,43]. In fact, one issue with this technique is that poor or variable sample preparation can lead to drastically different isotherm profiles and inconsistent data analysis [37]. Sample concentrations must satisfy the *c*-value window specifications as they impact the isotherm shape and the trend reaching saturation. As mentioned above, while using ITC, it must be assured that the thermodynamic assumptions are valid. One of the most common errors in ITC data analysis is the failure to allow signal equilibrium, as evidenced by the return to baseline before the next injection [33,36]. The fact that ITC data analysis cannot overlook the non-ideality of the macromolecules remains unavoidable; thus, precise experimental conditions need to be included in the report [36]. It should also be highlighted that studying weak interactions, *K_a_* < 10^3^ M^−1^ [31], remains challenging using ITC [33]. Although ITC results show whether an interaction is happening with minor enthalpy change, entropy-dominated interactions cannot easily be studied using this method [31].

Fluorescence Spectroscopy: PBUT accumulation in the blood compartment can cause protein conformational changes that may affect calorimetric results [32]. Fluorescence spectroscopy has been widely used to investigate ligand binding [44,45] and changes in protein tertiary structure during these events [32]. The fluorescence wavelength is sensitive to alterations in the immediate environment of the fluorescent probe. The high signal-to-noise ratio afforded by low amounts of fluorescent probes and rapid data acquisition is why this technique has been used for thermodynamic and kinetic studies of molecular interactions [46]. Moreover, the problematic requirement for a fluorescent probe is overcome by the presence of intrinsic fluorescence originating from the aromatic amino acids already present within the protein. Although there are three intrinsically fluorescent amino acids in HSA [46,47], tryptophan provides enough quantum yield and sensitivity for studying toxin–HSA interactions [46,48]. The fluorescence intensity emitted from tryptophan residue in HSA changes in the presence of toxins [47]. Although fluorescence optical techniques are fast, sensitive, and highly applicable in protein structure studies, their main limitation is their localized signal. The data given are limited to the environment close to the probe and the extent of its mobility. Therefore, the fluorescence spectra data are often contextualized using circular dichroism (CD), FT-IR, or NMR results. Moreover, fluorescence results do not explicitly describe pathways involved in conformational changes [46].

Fluorescence Quenching: The fluorescence quenching technique uses the intrinsic tryptophan fluorescence of proteins and provides data on the fluorophore location within a protein using quenchers that interact with external or internal tryptophan only [32]. The dependence of the emission intensity on quencher concentration (*Q*) is given by the Stern–Volmer equation (Equation (2)), where *F*_0_ and *F* represent the fluorescence intensity, $\tau_{0}$ and τ are fluorescent lifetimes, and the 0 subscripts show the absence of the quencher while the other parameters are the values in the presence of the quencher. *K_sv_* is the Stern–Volmer constant [44].

(2) $$\frac{F_{0}}{F}=\frac{\tau_{0}}{\tau }=1+K_{sv}\cdot Q$$

The association constant (*K_a_*) and the number of binding sites (*n*) can also be found by analyzing the quenching data [32]:

(3) $$\log\frac{F_{0\;}-\;F}{F}\;=n\log\;K_{a}-n\log\frac{1}{\left[Q\right]-\frac{\left(F_{0\;}-\;F\right)\left[P\right]}{F}}\;$$

Dynamic and static quenching modes can be clarified using the shape and position of fluorescence band changes with protein concentration, curvature, or linearity of the Stern–Volmer plots and order of magnitude for the Stern–Volmer constant [44,49]. These data can be used to distinguish between dynamic and static quenching on temperature and viscosity, affecting the fluorescence lifetime measurements [32,44]. Moreover, static quenching is observed when there is a strong coupling between HSA and toxins, where increasing the temperature causes a decrease in *K_sv_* [32].

Fluorescence probe displacement: This method is another form of fluorescence spectroscopy that detects changes in the fluorescence characteristics associated with probe binding to macromolecules and assists in identifying the binding location and potential competition in binding events [14,45,50,51]. The probes can possess either single or two binding sites and are expected to bind to specific locations on proteins (e.g., HSA). It is suggested that the concentration of ligands in the sample be as low as possible to avoid non-specific site occupation. This technique highly depends on the availability of binding sites on HSA. When the ligand concentration is high enough for binding sites to be saturated, interpretation of fluorescence changes in terms of individual binding sites is rather difficult. Moreover, several unrelated events, such as competitive displacements, might affect the probe’s fluorescence signal. The percentage of the probe’s displacement correlates with the fluorescence of the probe and HSA with and without the ligand present (*F*_2_ and *F*_1_, respectively), as shown in Equation (4)) [45]:

(4) $$\frac{F_{1}-F_{2}}{F_{1}}\times 100$$

The binding constant of HSA and toxin can be further obtained from the relation between the probe displacement and the fraction of sites occupied [49].

A decrease in probe–HSA complex fluorescence in the presence of the toxin is evidence that the toxin replaces the probe, and as the probes are site-specific, the results can provide an insight into the probable region that the toxin has an affinity on HSA. Warfarin and dansylsarcosine (DNSS) are generally used to locate uremic toxin binding on site I and site II, respectively. For instance, IS and IAA presence in the warfarin–HSA complex had an insignificant impact on the detected fluorescence intensity, as these two toxins have a higher affinity for site II [14,51]. Notably, the association and capacity of toxin to bind to HSA must be higher than that of the probes to result in a detectable change in fluorescence intensity. As HA interaction with HSA is relatively weaker on both sites, insignificant changes were observed using warfarin and DNSS as the probe [14].

Circular Dichroism (CD): If a substance absorbs circularly polarized light, either right-handed or left-handed, it exhibits circular dichroism [52]. CD is often used to determine protein secondary structure [32,38,42,53] and is highly accurate for α-helices; however, β-sheets, β-turns, and random coil contents are also obtained [52]. Changes in the CD spectrum as a function of ligand concentrations can be achieved and analyzed using widely available software that has been extensively used and reviewed [32]. However, one of the main problems with using CD for these PBUT studies is sample preparation, including the effect that salts have on the CD spectra [52,54].

On the CD spectrum of free HSA, the α-helical structure is indicated by two minima at 208 and 222 nm. In case of binding or presence of uremic toxins and HSA conformational changes, the mean residue ellipticity at each minimum may decrease, showing that the α-helicity is reduced. This trend was observed for CMPF, IAA, IS, and HA [38]. The relative reduction in helical content of HSA could be as high as 20.50% in the presence of IS [38]. Furthermore, CD was also utilized to observe HSA conformational changes with temperature and pH [42,55].

Nuclear Magnetic Resonance (NMR): NMR has been used to study ligand binding and macromolecular structure [56]. Compared to ITC, NMR gives a broader view of the dynamic of bindings at equilibrium and whether the interaction occurs on specific sites or solely causes HSA conformational changes without being attached [32,57]. In its infancy for studying PBUT interactions with proteins, 2-D NMR monitors all binding sites on the protein and quantitatively measures site-specific binding constants [58,59]. So far, only Li et al. [32] have utilized this technique to identify potential secondary and tertiary structure changes in HSA while interacting with PBUTs using STD (saturated transfer difference) NMR to detect the intensity of bindings through an on-and-off induced magnetic field without affecting the unbonded toxins. Not only could they recognize the binding percentage of PBUTs to HSA, but they could also observe the positional characteristics during the events. Li et al. utilized STD NMR to selectively transfer the induced magnetic field from HSA protons to those inbound IS and PCS without altering the free toxins in the solution; they could comprehend the strength of binding events from the resonance spectra and integrated area for each proton [32]. Furthermore, NMR is applicable in identifying specific sites on HSA where modifications can be performed to reduce the affinity of a compound [58], information that may be useful in designing effective competitors to prevent PBUT bindings to HSA.

Other Methods: Early research on PBUT binding to HSA utilized less sensitive or advanced mechanisms: equilibrium dialysis [14,19,60], ultrafiltration [14,28], Fourier-transform infrared spectroscopy (FT-IR) [47,61], and high-performance liquid chromatography (HPLC) [19,51]. X-ray crystallography has been used to further understand the binding location but is not widely used due to its availability, requirement to form crystals, and cost [50,62].

Equilibrium dialysis and HPLC measure a ligand’s binding to a macromolecule using separation. While both methods measure the clearance of free compounds from a ligand–protein solution, two chambers and separation membranes are often involved. Although equilibrium dialysis has been a preferred method for identifying plasma bindings, it is time-consuming and challenging to automate [56].

The rate-of-dialysis technique proposed by Kragh-Hanson [63] has made this technique’s performance more convenient. Still, it requires sample radio-labeling, and the working principle of this technique is to correlate the rate of transfer of the labeled ligand to the unbound fraction of ligands. In contrast, in HPLC, the mass transfer happens in microcolumns as measured using a differential mass detector [56,64,65]. HPLC is usually combined with spectroscopy techniques such as UV–vis [19] and fluorescence [51] in PBUT-HSA studies. Therefore, it has higher precision and could be automated to run relatively fast [65].

Ultrafiltration is a rapid method of measuring the binding extent of ligands to proteins [14]. However, binding constants cannot be quantified [56]. The non-specific binding (*NSB*) of toxins to HSA can be obtained by measuring the initial concentration of a ligand (*C_D_*) and that of it after ultrafiltration (*C_F_*) [19]:

(5) $$NSB=\frac{C_{D}-C_{F}}{C_{D}}$$

The applications of molecular docking tools in binding studies of HSA and toxins are also worth mentioning [35,42,62,66,67]. Although they involve specific assumptions and protocols depending on the working algorithms, molecular docking and molecular dynamic simulations have not only assessed researchers to obtain insight into the ligand–protein binding and complex structures but also provided tools for understanding the PBUT chemistry, production, and physiology [35,66]. A remarkable dataset on HSA residues participating in binding events for IS, PCS, IAA, and HA is provided using molecular dynamic simulation [62]. In more recent work, the same information for hydrophobic and hydrophilic residue receptors on HSA is given via molecular docking studies [66].

Amongst the methods mentioned above for obtaining the binding thermodynamics, association constants, and stochiometric data, ITC is often used [32,35,37,38,40,55]. Although ultrafiltration is as common, it requires separative membranes, while ITC allows affinity determination in a free medium [40]. Differential scanning calorimetry can be used in combination with ITC to obtain information on the thermostability of the protein [35,38,56]. Investigating the structure of HSA and evaluating the location of PBUT binding can be performed using NMR [32]; nevertheless, CD and fluorescence techniques are commonly employed due to their flexibility and affordability [14,28,32,35,38,42,45,50,51,55,68].

## 3. Insights into PBUT-HSA Interactions

Thermodynamic and kinetic parameters for PBUT binding with HSA differ based on the type of toxin involved and the interaction site (Table 2). Differences in HSA’s free fatty acid content, sample preparation methods, temperature, and salt concentration of the buffer could be some of the reasons for these differences [14]. The salt concentration in the buffer can have a significant impact because changes in ionic strength are observed to substantially affect the binding mechanism and potentially alter the electrostatic forces involved [15,37,62]. 3-carboxyl-4-methyl-5-propyl-2-furan propanoic acid (CMPF), phenylacetic acid (PAA), indoxyl sulfate (IS), indole 3-acetic acid (IAA), p-cresyl sulfate (PCS), and hippuric acid (HA) (Figure 1, [14]) have been shown to have a high affinity for at least either site I or II on HSA, if not for more locations depending on solution concentration [28]. Binding mechanisms are determined from changes in thermodynamic parameters (H, S, and G): negative ΔH indicates an exothermic event arising from electrostatic interactions, and negative ΔS suggests the involvement of hydrogen bonding [31,38].

3-carboxyl-4-methyl-5-propyl-2-furan propanoic acid: CMPF is a strongly lipophilic urofuranic acid that exclusively binds to site I and is known to impact the elimination of bilirubin and thyroxine, affecting the progression of liver damage and cardiovascular disease [18,50,69,70]. In CKD patients, the buildup of CMPF in the blood inhibits renal elimination of other compounds [70], including inhibiting warfarin and dansylsarcosine (DNSS) binding to HSA [14]. CMPF binding to HSA is exothermic, arising from both electrostatic interactions and the hydrophobic effect. The latter is thought to be due to bulky hydrophobic dicarboxylic acid groups on CMPF [38,50]. HSA binding of CMPF is pH dependent; pH increase leads to a change in the charge properties of HSA, altering its confirmation and yielding an electrostatic repulsion that impedes CMPF binding [19,50]. HSA binding of CMPF is also mildly sensitive to increased temperature [19].

Based on the fluorescence displacement data, CMPF and Bilirubin compete for the same site on HSA, with association constants of 130 × 10^5^ and 500 × 10^5^ M^−1^, respectively [14,50]. Moreover, prostaglandin shares the same binding site as CMPF on HSA as an endogenous substance [14]. However, the association constant obtained by ITC in a much more recent study is 5.76 × 10^6^ M^−1^ on a higher affinity site [38]. Observed thermodynamic parameters differed between the two studies of CMPF-HSA interaction [19,38]. The significant difference observed was that the older work using equilibrium dialysis (37 °C) showed an entropy increase, whereas subsequent work using ITC (25 °C) found that entropy decreased. It has been reported that this temperature difference likely is not the dominant reason for the change in entropy [19].

CMPF is preferentially bound by Sudlow’s site I (subdomain IIA), whereas other PBUTs (PCS, IS, IAA, and HA) have a higher affinity for Sudlow’s site II (subdomain IIIA). Zaidi et al. [38] showed that among IAA, HA, and IS, the latter has the highest affinity for this site. Due to their carboxylate group, IAA and HA have stronger electrostatic interactions on site II than IS and PCS [62]. However, HA and IAA are replaced by IS and PCS on the high-affinity site in higher concentrations of these four toxins in healthy serum and hemodialysis patients’ serum samples [62,71]. Devine et al. suggested that investigating the binding mechanism for purified HSA may overlook the involvement of other competitors, such as fatty acids, as they showed that the IS association constant to human plasma is 7.46 × 10^4^ M^−1^, much less than the IS affinity for HSA (1.17 × 10^6^ M^−1^) on only one independent binding site [15]. However, experimenting with native plasma will complicate the sample preparation and locating the binding site on proteins.

Indoxyl Sulfate: IS retention in the blood compartment is correlated to the progression of CKD and many co-morbidities [4]. IS binding to HSA mainly occurs at site II, is exothermic, and involves electrostatic and hydrophobic interactions [18,32,40,62]. The interaction is enthalpy-driven, as the association constant of IS-HSA decreases with increasing temperature; it is also counterbalanced by entropy [15,40]. Entropy changes are considered one of the main factors involved when IS competes with other uremic toxins, especially PCS, for binding site II [40]. Based on ITC results, Bergé-Lefranc et al. claimed that IS interaction with HSA follows “enthalpy-entropy compensation”, as they showed a linear relation between ΔH and TΔS at four different temperatures, which is also attributed to the heat capacity change in HSA [40]. Although the HSA conformational changes highly affect the likelihood of bindings, Yu et al. showed that the binding affinity for IS does not significantly change after urea-induced carbamylation of HSA [37]. Observing IS binding to HSA in both sites I and II showed that the binding constants seem relatively insensitive to pH changes [60]. However, this is in contrast to later work that shows IS has a weaker attachment to HSA in acidic and alkaline conditions [55]. Nevertheless, these different association constants for the IS-HSA complex may result from HSA conformation changes in these extreme pH domains, viz., lower than five or higher than ten. That said, using pH changes to alter the binding of toxins may contribute to freeing IS from HSA but likely will have deleterious effects on blood cells and other proteins [55].

Indole 3-acetic Acid: IAA is an essential metabolite for tryptophan and competes with that for binding sites on HSA [72]. IAA binding to HSA is exothermic, with negative and positive ΔS on HSA sites I and II, respectively, and is slightly dependent on pH with the same trend as IS. The primary binding site is considered site II with relatively lower affinity and effect on HSA’s secondary structure than IS [38,55]. Although different methods were used to obtain the IAA association constant with HSA, they correlated with sites I and II on HSA [14,38]. Nevertheless, other thermodynamic properties are only reported in Zaidi et al.’s work with ITC and deviate from the molecular docking results, specifically on lower affinity sites [14,38,66].

p-Cresyl sulfate: Up to 90% of PCS is bound by blood proteins and has been correlated to all-cause mortality and the progression of renal damage in patients regardless of dialysis treatment [18,51]. PCS and IS demonstrate competitive interactions with HSA and are often compared thermodynamically [32,40]. IS displacement by PCS is very likely on site II at a PCS concentration of 200–1000 µM, which is relevant to the CKD patients’ conditions at the last stages [51]. PCS binding to HSA is exothermic with a decrease in entropy (negative ΔS), indicating that electrostatic forces are involved [32]. Although IS and PCS share the same anionic group, sulfate, PCS forms more hydrogen bonds with HSA on site II [62]. At higher concentrations, PCS may displace IS, accounting for the fluctuation of free PCS concentration in the clinical data of patients under hemodialysis [51]. PCS binding to HSA slightly depends on pH, similar to IS and IAA [55].

Hippuric Acid: HA accumulation causes renal tubule cell damage and advances CKD progression, inhibiting HSA interactions with other compounds [18]. There are few articles on HA interactions with HSA, yet the works reporting HA binding association constants using ITC and equilibrium dialysis are within the same order of magnitude [14,35]. It is worth mentioning that HA has an affinity for both sites on HSA, but its free form is highly accumulated in the blood [14], as it has the highest hemodialysis reduction among the other PBUTs (Table 1). HA binding to HSA is exothermic and involves electrostatic interactions and hydrogen bonds; however, changes in ΔH dominate the binding event occurrence [35,38]. Even though HA binds to site II with higher affinity than site I [35], Davilas A. et al. showed that HA, as well as IS and IAA, reduces the bonded fraction of other drugs, such as diflunisal, to site I on HSA [13,14]. It is worth noting that although HA interaction with HSA is weaker than IS and IAA, it could displace them on HSA site II in high concentrations [14]. Nevertheless, the number of studies reporting the binding parameters is fewer than IS and IAA, which share the same binding site on HSA.

Phenylacetic acid: PAA binding to HSA is exothermic, but the enthalpy change upon binding is relatively less than other PBUTs, suggesting a small hydrophobic effect that may be due to the aromatic structures in the toxin and weak electrostatic forces [37]. Although PAA induces oxidative stress and systematic inflammation that advances CKD progression [18], there are limited studies on thermodynamics and binding mechanisms to HSA. So far, it is known that PAA has relatively less affinity for both sites on HSA, suggesting there might be another site of interaction where it has been claimed that the binding events are sequential on two regions of HSA. Regarding the main binding sites on HSA, as CMPF displaces PAA on site I, binding to site II is tight enough that it is not replaced by either IS or PCS, even though the concentrations of the later toxins are significantly higher than PAA in CKD patients [28,37]. Further data suggest that other plasma proteins may be involved in binding PAA [28].

## 4. Insights into Indoxyl Sulfate and p-Cresol Sulfate Binding to HSA

Despite more publications for IS and PCS binding to HSA, the resulting high-affinity *K_a_* varies as much as three orders of magnitude. It has been postulated that ultrafiltration results differ due to the chloride ion content of the buffers used, where chloride ions reduce drug binding to HSA, which are effects that may account for lower *K_a_* for PCS and IS using chloride-free buffer [14,51]. Moreover, Bergé-Lefranc et al. suggested that the significant differences in the values obtained for IS are mainly due to the method used, in their case, ITC. They claimed that separative membranes in ultrafiltration obstruct the results as separation methods also lack efficacy in hemodialysis membranes [40].

Studying the STD NMR spectra for IS and PCS interactions with HSA, Li et al. claimed that 32.5% and 23.8% of the molecules participated in the event, respectively. Regarding the IS structure, it was shown that the sulfated group is probably responsible for the high affinity to HSA. Comparing the behavior with the PCS binding event, they proposed that PCS-HSA formation requires a minor “steric hindrance” and results in a more homogenous complex concerning the binding site [32]. Furthermore, STD NMR results showed that ibuprofen or warfarin could displace IS as the IS-HSA signal intensity decreases. These two drugs bind to both Sudlow’s sites, suggesting that IS has a relatively strong affinity for both. PCS displacement with ibuprofen was relatively lower, and no significant signal changes were observed by the introduction of warfarin in the NMR signal intensity for this toxin. Although this contrasts with the same work’s ITC results, it suggests that there may be another site on HSA, which is a novel finding [32]. Earlier work using warfarin as a displacement probe also claimed that PCS is unlikely to bind to HSA site I, as its presence did not affect the signal intensity [51].

Although on the higher affinity binding site IS and PCS are shown to have comparable *K_a_* values, their binding to HSA is competitive, where introducing either of them to a preformed HSA complex leads to an increase in the free fraction of that previously bound toxin [51]. If PCS does not bind to HSA on site I, the presence of IS is likely compromising PCS binding on an unknown site on HSA.

## 5. Competitive Binders to Inhibit HSA Uptake of PBUTs

Engineering competitive binders to enhance PBUT clearance from the blood compartment relies on a thorough understanding of how they interact with proteins. HSA is the predominant protein due to its concentration and propensity for binding small molecules. Two main routes can accomplish this outcome: i. exploring molecules that displace toxins from HSA so that they can be cleared using membrane-based dialysis techniques and ii. building surfaces that have higher affinities for these PBUTs than HSA. Herein, we will summarize the significant work on developing molecules that displace toxins from HSA.

Some of the earliest work on competitive binders for CMPF to HSA proposed that drugs with two carboxyl groups and enough hydrophobicity, such as methotrexate, may be an active binder for HSA to displace CMPF [50]. This was based on understanding how bilirubin and CMPF bound to HSA, where the two carboxyl groups and the distance between them were thought to be vital to their binding. In addition to these physicochemical properties of potential competitive binders, the effect of concentration and HSA site saturation must be considered. For instance, o-methyl red and CMPF share the same binding site on HSA, the latter having a higher affinity at low but not higher concentrations; when both are at 200 µM, o-methyl red is nearly 80% bound, compared to only 70% for CMPF [19].

Ibuprofen and warfarin are often considered for displacing PBUT from HSA [14,32,51]. Although Li et al. observed that these two substances could displace IS and PCS on higher affinity sites from ITC, NMR showed that only ibuprofen had this capability [32]. High removal of IS and PCS via ibuprofen infusion to hemodialysis patients also showed the enhanced dialytic removal of these two toxins due to its association constant of 1 × 10^6^ M^−1^ on site II of HSA, which is 10 times higher than those for PCS and IS [51,73]. It is noteworthy that these findings were the first clinical study on ibuprofen. Nevertheless, ibuprofen infusion also displaces albumin-bound tryptophan and lacks the ability to improve the clearance of PBUTs with high affinities to site I [73].

Free fatty acids have also been shown to have the potential to significantly decrease HSA binding of IS, PCS, and IAA and hence increase the efficacy of hemodialysis [74]. However, as CMPF holds the highest binding percentage compared with the named toxins, the increase in the unbound fraction for this toxin in the presence of free fatty acids was insignificant [74,75]. Non-esterified fatty acids, such as octanoate and docosahexaenoic, can lead to total displacement of IS and PCS at a concentration of 0.24 M. These substances strongly interact with HSA and displace uremic toxins bound to site II in in-vitro studies; however, they are toxic and enhance cardiovascular complications and oxidative stress [76].

Thus, the question remains how to design competitive binders that do not have deleterious effects if they are also not cleared. Especially considering that they are bound to HSA so firmly that they cannot be removed from the blood compartment. Additionally, the goal must be to have the least number of molecules introduced to displace the maximum amount of PBUTs from HSA to enhance their clearance and minimize potential adverse health outcomes [74]. Davilas et al. pointed out that while exploring various binding competitors, one must consider that in vivo HSA is surrounded by multiple endogenous and exogenous substances competing for binding, and simulating the same condition to evaluate a specific binder is often problematic [13]. Ideal binding competitors’ characteristics include the ability to block both sites on HSA, which are the leading region for PBUT interactions, minimize therapeutic consequences, and utilize clearance pathways available during kidney dysfunction [73]. Perhaps the path forward lies in developing binders that can be consumed by the surrounding cellular milieu to effectively remove them from the blood compartment or that they can be easily displaced from HSA with a minor modification in environmental conditions during dialysis itself.

## 6. Conclusions

Accumulation of uremic toxins in the blood compartment of CKD patients advances kidney dysfunction and various co-morbidities. The lack of efficacy in hemodialysis for clearing PBUTs highlights the importance of investigating the binding affinity of PBUTs with blood proteins. Human serum albumin has two prominent locations for binding PBUTs. Herein, we summarized the data on binding mechanisms, sites, thermodynamic parameters, and methods used to capture these data to highlight the design criteria for advancing PBUT removal through engineering competitive binders. These competitive binders could act through targeted adsorption of PBUTs from the solution phase or by displacing them from the binding sites found on proteins such as HSA. However, the binding mechanisms of many uremic toxins to plasma proteins are unclear. Common techniques used to understand these interactions are summarized here; advanced calorimetric and spectroscopic techniques are understudied in toxin–protein interactions, e.g., vibrational spectroscopy, and may help achieve valuable insights into the nature of binding. Moreover, the available data on the phenomena in living systems are significantly limited. Amino acids and endogenous molecules surrounding the HSA in the blood compartment largely influence the binding mechanisms. Investigating the sole HSA and toxin interaction omits these side competitions for binding. Molecular docking applications could be one of the theoretical approaches to simulate the in vivo conditions and inform the molecular design for competitive binders. Finally, if multiple competitive binders are added to the blood compartment to dislodge uremic toxins from proteins, such as HSA, there is a risk that they themselves will have deleterious effects on the host. Thus, these binders may need to be designed to remove the maximum number of PBUTs whilst being consumed by localized cellular milieu. Most binders proposed so far are exogenous drugs, and their clearance pathways are still not thoroughly discussed in the literature.

## Figures and Tables

**Table 2:** Thermodynamic properties of PBUT-HSA binding for CMPF, IS, IAA, HA, PAA, and PCS with sample properties and conditions.

Toxin	Protein Buffer	pH	T (°C)	Methodology	Results						Discussed Competitors	Ref.
					Binding Site	*K_a_* (M^−1^)	ΔH (kcal mol^−1^)	TΔS (kcal mol^−1^)	ΔG (kcal mol^−1^)	*n*		
**CMPF**	**HSA** **Sodium Phosphate**	**7.4**	**25**	**ITC,** **Far-UV CD, FT-IR, X-ray crystallography,** **DSC**	**High affinity** **Low affinity**	5.76 × 10^6^ 4.57 × 10^3^	−12.89 −14.89	−3.70 −10.21	−9.19 −4.68	-	-	[38]
	HSA (defatted) HSA modifications: HNB-HSA, TNM-HSA, SA-HSA, DEP-HAS Sodium phosphate (dibasic)	6.8–8.2	25	X-ray crystallography, fluorescence quenching, and probe displacement	Site I	130 × 10^5^	-	-	-	1	Bilirubin, warfarin, phenylbutazone	[50]
	HSA BSA Isotonic sodium phosphate-chloride	6.4–8.3	10, 25, 37	Equilibrium dialysis, HPLC	High affinity (at 37 °C, pH 7.4) Low affinity (at 37 °C, pH 7.4)	4.8 × 10^6^ 0.6 × 10^3^	(Ref data, 25 °C) –2.15	4.78	−6.93	0.6 10.3	o-methyl red	[19]
	HSA (defatted) Deionized and distilled water, phosphate buffer	7.4	25	Fluorescent probe displacement, ultrafiltration, equilibrium dialysis	High affinity Low affinity	130.5 × 10^5^ 33.4 × 10^4^	-	-	-	1 2	Warfarin, DNSS	[14]
**IS**	HSA (defatted) Deionized and distilled water, phosphate buffer	7.4	25	Fluorescent probe displacement, ultrafiltration, equilibrium dialysis	High affinity Low affinity	16.1 × 10^5^ 8.3 × 10^3^	-	-	-	1 3	Warfarin, DNSS	[14]
	HSA Phosphate buffer	7.2	25, 30, 37	ITC, fluorescence quenching, CD, STD NMR	Overall interaction with HSA (at 25 °C, pH 7.2)	10.06 × 10^3^	−16.8	−11.96	−4.81	1.08	Ibuprofen, warfarin	[32]
	HSA Phosphate buffer	7.4	25	HPLC, fluorescent probe displacement	High affinity Low affinity	0.98 × 10^5^ 8.0 × 10^3^	-	-	-	1 1.6	Warfarin, dansylsarcosine	[51]
	HSA (defatted) Deionized and distilled water Phosphate buffer	6.5, 7.4, 8.5	25	Equilibrium dialysis	High affinity (at pH 7.4) Low affinity (at pH 7.4)	9.1 × 10^5^ 0.8 × 10^3^	-	-	-	1 3	Warfarin, DNSA, DNSS	[60]
	HSA (defatted) Modified HSA morpholine-N-oxide	7.4	25, 30, 37	ITC	High affinity (at 25 °C and ionic strength 20—native HSA) Low affinity (at 25 °C and ionic strength 20—native HSA)	-	-	-	−7.55 −4.94	-	-	[37]
	Modeled—HSA			Molecular docking	Overall interaction with HSA	-	−7.5	-	-	-	Laccase 1KYA and 3FU9	[66]
	HSA Phosphate buffer	1.9–12.9	37	CD, ITC	Overall interaction with HSA (at pH 7.1)	1.27 × 10^5^	−20.23	−15.63	−7.25			[55]
	HSA 2-(N-morpholino) ethane sulfonic acid) buffer	7.4	15, 20, 25, 37	ITC	Overall interaction with HSA (at 25 °C)	1750	−8.65	−4.63	−4.35	0.98		[40]
	HSA Sodium phosphate	7.4	25	ITC, Far-UV CD, FT-IR, X-ray crystallography, DSC	High affinity Low affinity	1.04 × 10^6^ 6.59 × 10^5^	−20.05 −14.39	−11.80 −6.90	−8.25 −7.49	-	-	[38]
**IAA**	HSA (defatted) Deionized and distilled water, phosphate buffer	7.4	25	Fluorescent probe displacement, ultrafiltration, equilibrium dialysis	High affinity Low affinity	2.1 × 10^5^ 0.8 × 10^4^	-	-	-	1 5	Warfarin, DNSS	[14]
	Modeled—HSA			Molecular docking	Overall interaction with HSA	-	−6.8	-	-	-	Laccase 1KYA and 3FU9	[66]
	HSA in experiments Model samples—HSA			CD, SPR, molecular docking	Domain 2 Domain 3 Domain 2-3 HSA	5.92 × 10^3^ 4.65 × 10^5^ 1.25 × 10^5^ 2.75 × 10^4^						[42]
	HSA Sodium phosphate	7.4	25	ITC, Far-UV CD, FT-IR, X-ray crystallography, DSC	High affinity Low affinity	1.78 × 10^5^ 8.27 × 10^4^	−13.94 −3.8	−6.40 2.8	−7.45 −6.60	-	-	[38]
**HA**	HSA (defatted) Deionized and distilled water, phosphate buffer	7.4	25	Fluorescent probe displacement, ultrafiltration, equilibrium dialysis	High affinity Low affinity	0.1 × 10^5^ 0.3 × 10^4^	-	-	-	1 7	Warfarin, DNSS	[14]
	HSA Sodium phosphate	7.4	25, 30, 37	ITC, DSC, molecular docking, CD, fluorescence quenching	High affinity (at 25 °C) Low affinity (at 25 °C)	2.75 × 10^4^ 2.8 × 10^3^	−4.41 −7.58	1.90 2.88	−6.05 −4.69	0.97	-	[35]
	HSA in experiments Model samples—HSA			CD, SPR, molecular docking	Domain 2 Domain 3 Domain 2-3 HSA	9.52 × 10^3^ 5.78 × 10^4^ 9.26 × 10^3^ 2.11 × 10^4^						[42]
**PAA**	HSA (defatted) Modified HSA morpholine-N-oxide	7.4	25, 30, 37	ITC	High affinity (at 25 °C and ionic strength 20—native HSA) Low affinity (at 25 °C and ionic strength 20—native HSA)	-	-	-	−5.90 −4.04	-	-	[37]
	BSA HSA (with fatty acids)	7.4	37	Ultrafiltration, fluorescent probe displacement	High affinity Low affinity	3.66 × 10^5^ 3.86 × 10^4^				0.07 1.83		[28]
**PCS**	HSA Phosphate buffer	7.4	25	HPLC, fluorescent probe displacement	High affinity Low affinity	1 × 10^5^ 19.6 × 10^3^	-	-	-	1 1.2	Warfarin, dansylsarcosine	[51]
	Modeled—HSA			Molecular docking	Overall interaction with HSA	-	−6.3	-	-	-	Laccase 1KYA and 3FU9	[66]
	HSA Phosphate buffer	7.2	25, 30, 37	ITC, fluorescence quenching, CD, STD NMR	Overall interaction with HSA (at 25 °C, pH 7.2)	1.39 × 10^3^	−27.5	−23.29	−4.23	1.03	Ibuprofen, warfarin	[32]

## Data Availability

Not applicable.
